# Stem Cell Therapy for Degenerative Disc Disease

**DOI:** 10.1155/2012/961052

**Published:** 2012-04-24

**Authors:** Doniel Drazin, Jack Rosner, Pablo Avalos, Frank Acosta

**Affiliations:** ^1^Department of Neurosurgery, Cedars-Sinai Medical Center, Los Angeles, CA 90048, USA; ^2^Regenerative Medicine Institute, Cedars-Sinai Medical Center, Los Angeles, CA 90048, USA

## Abstract

Low back pain is widely recognized as one of the most prevalent pathologies in the developed world. In the United States, low back pain is the most common health problem for adults under the age of 50, resulting in significant societal and personal costs. While the causes of low back pain are myriad, it has been significantly associated with intervertebral disc (IVD) degeneration. Current first-line therapies for IVD degeneration such as physical therapy and spinal fusion address symptoms, but do not treat the underlying degeneration. The use of tissue engineering to treat IVD degeneration provides an opportunity to correct the pathological process. Novel techniques are currently being investigated and have shown mixed results. One major avenue of investigation has been stem cell injections. Mesenchymal stem cells (MSCs) have shown promise in small animal models, but results in larger vertebrates have been mixed.

## 1. Introduction

### 1.1. IVD and Low Back Pain

Intervertebral discs act as the main joints of the spinal column, providing both stability and flexibility. In addition to facilitating bending, flexion, and torsion, they also help to transmit loads applied to the spine. In the normal course of aging, the intervertebral disc (IVD) and in particular the nucleus pulposus (NP) undergo extensive morphological and cellular changes resulting in hardening of the NP and a decrease in structural integrity, disc height, and flexibility of the IVD as a whole [1, 2].

Low back pain has been strongly associated with such IVD degeneration [3, 4]. Numerous epidemiological studies suggest that such back pain is widespread, frequently debilitating, and costly. Approximately 25% of American adults reported low back pain occurring in the past 3 months. This corresponds to over 54 million individuals [5]. Over a lifetime, 70% to 80% of people will at some time experience back pain [6]. Accordingly, in the United States, low back pain is the fifth most common reason for physician visits, constituting approximately 2.3% of all appointments [5, 7].

Low back pain is frequently debilitating and as such is responsible for significant productivity losses. Accounting for 149 million lost work days annually, back pain is the second most common reason for sick leave, behind only the common cold [8–10]. In a given year, 8% of the entire working population will be disabled by back pain [11]. This results in significant economic losses. As of 1997, it was calculated that back pain resulted in an aggregate productivity loss of $28.17 billion in the US, although by some estimates this figure may have been as high as $87.8 billion [12, 13].

At least one recent study suggests that the incidence of low back pain is increasing. Freburger et al. [14] found a 6.3% increase—from 3.9% to 10.2%—in reported chronic low back pain between 1992 and 2006. Given the costs and discomfort associated with chronic low back pain, this increase is concerning and underscores the import of exploring new treatment modalities.

In this paper, we discuss the potential of using MSCs to treat IVD degeneration. We comment on current research and conclude with recommendations for further study.

## 2. The IVD: Structure and Degeneration

### 2.1. Disc Morphology

**Figure 1:**
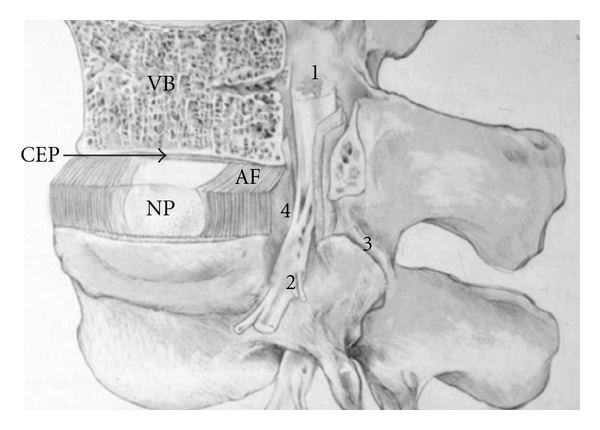
Illustration of the main intervertebral disc structures and vertebral column. CEP: cartilage endplate; AF: annulus fibrosus; NP: nucleus pulposus; VB: vertebral body; 1: spinal cord; 2: nerve root; 3: apophyseal joint; 4: site of NP protrusion and nerve root compression after IVD degeneration.

The IVD is avascular and consists mainly of a macromolecular extracellular matrix (ECM) with a low-density population of cells that help to maintain this ECM. Grossly, a normal IVD consists of a central NP surrounded by the annulus fibrosus (AF), all of which is sandwiched between two cartilaginous endplates (EPs) (Figure 1). The NP is relatively fluid, composed primarily of an ECM of collagen type II and proteoglycans. Functionally, the collagen imparts tensile strength, while the proteoglycans attract and bind water, providing resilience to compression. Suspended throughout this ECM are chondrocyte-like cells. Commonly, the consistency of the NP is described as “gel-like.” In turn, the AF is composed of a series of concentric rings (lamellae) which are primarily collagen I. The high-percentage of collagen makes the AF rigid, a property that helps it to contain the more fluid NP and contribute to the integrity of the disc. Finally, the endplates separate the NP and AF from the adjacent vertebral bone.

### 2.2. The Aging Disc

Histologic assessment has shown that disc degeneration definitively begins in the early teenage years [2, 15]. The discs of the lumbar spine bear a disproportionate amount of this wear [2]. Far from being static, the disc ECM is subject to continuous synthesis and degradation [16]. In IVD degeneration, the rate of matrix anabolism slows, while matrix catabolism increases. This results in a number of changes. Proteoglycan content in the NP drops significantly, and with it the ability of the ECM to attract and retain water [16]. The number of chondrocytes in the ECM decreases [15, 17]. Macroscopically, fibrous tissue forms in the NP, resulting in a loss of gel-like character and ultimately leading to a dissolution of the distinction between NP and AF [2]. Repeated mechanical loading [1, 18] and declining nutrition [1, 19, 20] have been implicated as the two most critical factors in degeneration.

Mechanical overloading of the IVD has been shown to induce catabolic activity associated with degeneration [21]. It has also been suggested that the routine cycle of disc deformation and recovery caused by normal activity could eventually lead to fatigue failure of the disc [1].

Insufficient nutrition is significant in slowing matrix anabolism. Because the IVD is avascular, it must receive nutrients through diffusion. Blood vessels terminate at the EP and nutrients then move down concentration gradients across the plate and through the ECM to reach embedded cells. It is well established that the EPs become less permeable with age [20, 22], and Boos et al. [15] found histologic evidence that a decrease in endplate blood vessels coincides with an increase in disc ECM breakdown. Studies on disc nutrition have suggested that glucose is the critical nutrient for maintaining cell viability, with oxygen and pH acting as secondary factors [19, 23]. When nutrition of the disc is sufficiently impaired, disruption of matrix synthesis and cell death can occur [24, 25].

The other component in disc degeneration is breakdown of the matrix. Matrix metalloproteinases (MMPs) and aggrecanases are two classes of enzymes involved in both normal matrix turnover and degeneration. These enzymes degrade the components of the ECM and have been found at elevated levels in degenerated discs [26, 27].

## 3. Treatments: Present and Future

### 3.1. Current Treatments

Current treatments for disc degeneration fall into two categories. Conservative, nonsurgical management entails analgesics, rehabilitation programs, and lifestyle adjustments such as weight loss. Surgical intervention involves spinal fusion and disc arthroplasty [28, 29]. While conservative management is the preferred treatment method for most cases of IVD degeneration, patients not benefiting from such management can realize benefits from surgical fusion [30]. Nevertheless, neither conservative nor surgical management addresses the underlying process of IVD degeneration, and for many patients neither is effective at relieving low back pain. Furthermore, fusion surgery has significant downsides. Beyond the loss of flexibility between fused vertebrae, fusion can also increase stress and strain on adjacent discs and thus accelerate their degeneration, necessitating further surgical intervention [31–33].

### 3.2. Emerging Treatments

**Figure 2:**
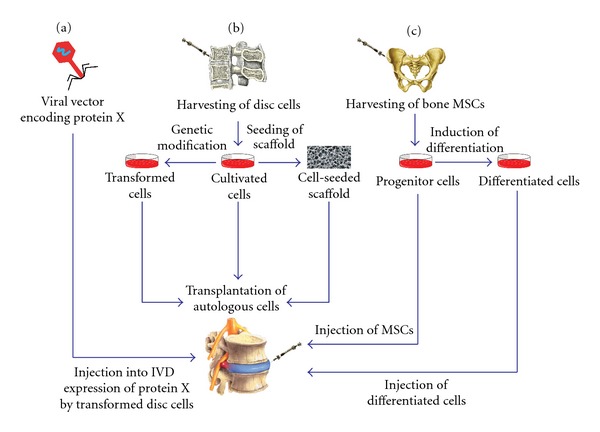
Different treatments for IVD degeneration are illustrated. (a) Injecting a viral vector into the IVD causes expression of the coded protein by the transformed disc cells. (b) Cells from the NP are harvested and then can be cultivated, genetically modified, or seeded into a scaffold before being transplanted into the IVD. (c) Bone MSCs are harvested and injected into the IVD as MSCs or as differentiated cells.

A growing understanding of the molecular changes associated with IVD degeneration has led to a burgeoning exploration of various treatments designed to directly address these changes [17]. In recent years, therapies targeting several molecular and cellular aspects of degeneration have been explored. One approach has been the direct injection or stimulation through gene therapy of a number of growth factors involved in regulating matrix anabolism [34, 35]. This technique has shown promising results *in vitro *and *in vivo* in small animal models [36–40]. Another major avenue of investigation has been cell therapy. The goal of cell therapy is to increase ECM synthesis by repopulating the degenerate NP. To accomplish this, one of several types of cells is injected directly into the NP (Figure 2). Cell types utilized thus far include NP cells [41–43], chondrocytes [44–46], and MSCs [44, 47–55], all of which have exhibited potential for slowing and repairing degeneration. In this paper, we focus on research regarding MSCs.

## 4. Mesenchymal Stem Cells

### 4.1. Background and General Therapeutic Use

MSCs are undifferentiated cells found in several adult tissues. The multipotent nature of individual MSCs was first demonstrated by Pittenger et al. [56], and since then they have been found to be pluripotent, giving rise to endoderm, ectoderm, and mesoderm cells [57]. MSCs are well suited to therapeutic application because they can be easily cultured and have high *ex vivo* expansive potential [58]. They are also capable of robust, persistent engraftment [57]. Furthermore, use of MSCs avoids the ethical issues raised by embryonic stem cell harvesting [59, 60]. MSCs have shown therapeutic promise in a number of diverse applications including regenerating infracted myocardium [61, 62], improving functional recovery from ischemic stroke [63], and rescuing liver failure [64].

A number of mesenchymal tissues have been investigated as MSC sources in adults (Table 1). Chief among these are bone marrow [56], periosteum [65], synovial membrane [66], and adipose tissue [67]. Two recent studies have suggested that MSCs isolated from different tissues exhibit different levels of expandability, chondrogenesis, osteogenesis, and adipogenesis, with synovium-derived MSCs being generally superior [68, 69]. 

### 4.2. Use in IVD Degeneration: *In Vivo* Studies

A number of *in vivo *studies have examined the use of MSCs to slow the process of IVD degeneration and regenerate the matrix. In 2003, Sakai et al. [51] conducted the first study exploring the use of MSCs to repair IVD degeneration *in vivo *using a rabbit model. Partial aspiration of the NP was used to induce degeneration, and autologous MSCs embedded in an Atelocollagen gel were then injected into discs. This procedure was found to prevent histological and morphological disc degeneration when compared to a nontreated, degeneration-induced control. Overall NP and AF structure, cell volume, and matrix formation (in particular proteoglycan content) were maintained up to 8 weeks after injection, and implanted MSCs were found to have differentiated into cells resembling original disc cells.

A number of other studies examining the use of MSCs in small animals have demonstrated the ability of these cells to survive, differentiate towards disc cells, and produce matrix components including collagen II and proteoglycans. This has been shown both with and without a number of cellular scaffolds (Atelocollagen gel, hyaluronan gel, and PuraMatrix) using autologous, allogenic, and xenogeneic (specifically, human) MSCs, and with follow-up times ranging from approximately one month to four months [47, 52–55]. Using a rabbit model, Zhang et al. [55] found that transplanted allogenic MSCs survived and increased proteoglycan and collagen II synthesis in the NP. Wei et al. [53] used a rat model to assess the ability of human MSCs to proliferate and function within the IVD. After 6 weeks, MSCs demonstrated survival and differentiation towards disc cells. Widespread success using allogeneic and xenogeneic MSCs may reflect the immune privilege of the IVD [70], as well as the immunosuppressive capabilities of MSCs [71].

While small animal models have yielded universally positive results, the results of large animal studies have been mixed. Henriksson et al. [48] injected human MSCs into porcine discs which were then harvested at up to 6 months. At followup, MSCs were found to have survived and differentiated toward disc cells, exhibiting matrix-producing functionality. Similarly, Hiyama et al. [49] found MSC injection into degeneration-induced canine discs to increase proteoglycan content and effectively mitigate degeneration. While these results are encouraging, another recent large animal study casts doubt on the potential of MSCs to treat IVD degeneration clinically. Acosta et al. [44] injected injured porcine discs with allogeneic MSCs. Discs were then harvested at 3, 6, and 12 months. At all followups, no viable MSCs or proteoglycan synthesis as observed. One reason postulated for this includes the larger disc size and therefore greater nutrient restriction present in the porcine model as compared to small animal models. This larger disc size more closely mimics the conditions in adult human IVDs, where nutrients must travel up to 8 mm from the terminal end of the blood vessel to cells in the center of the disc [23].

## 5. Future Directions

### 5.1. Critique of Current Studies

A notable criticism of current studies involving *in vivo* implantation of MSCs is that they do not accurately replicate the environment of the human degenerate disc. This is true for several reasons. Firstly, in all *in vivo* studies to date, MSCs have been implanted either into unmodified, healthy, young discs [47, 52, 53, 55] or into discs where degeneration was simulated by aspiration of the NP [44, 48, 49, 51] or annular injury [50, 54]. While these techniques have been shown to induce degeneration of the NP and AF as evidenced through MRI [72], there is no evidence that they lead to the EP damage typical in painfully degenerated IVDs, damage which likely impairs nutrient diffusion. Lack of nutrients has been found to impair ECM synthesis [24, 25] and poor EP permeability is highly correlated with morphologic and biochemical degeneration [73]. The central role of nutrition in the efficacy of MSC treatment is further implicated in the results of Acosta et al. [44], where it was hypothesized that the relatively larger discs of minipigs compromised nutrient diffusion and prevented the survival of implanted MSCs. Based on current research, it is unclear whether repopulation without nutritional supplementation will lead to effective matrix anabolism. In the future, development of a standardized *in vivo* model that more accurately mimics disc degeneration in humans would allow for more meaningful study of all therapies targeting molecular and cellular components of degeneration.

It is also worth noting that the histological and morphological slowing and reversal of IVD degeneration may not necessarily relieve low back pain. In fact, this was the outcome of one clinical study using MSCs to repair cartilage in osteoarthritis patients. Although biopsy and arthroscopic observation demonstrated new cartilage growth, no significant clinical improvement was reported [74, 75]. At present there exists no animal model for low back pain, making the therapeutic benefit of NP regeneration challenging to study [76]. The clinical benefit of restoring matrix integrity must be further explored.

### 5.2. Obstacles in Translation to Clinical Use

Before stem cells can be adequately and efficiently used in IVD degeneration, it is imperative that the mechanisms of pathogenesis are more clearly understood in order to answer many questions that have been left from previous studies. The absence of an animal model for low back pain involving IVD degeneration makes it difficult to truly study and assess the effectiveness of cell therapy. It has previously been studied that the degenerating IVD creates a harsh environment by decreasing nutrient supply from the EP, increasing the acidity of the microenvironment and elevated inflammatory substances [19, 74]. This hardly is the ideal environment required for a successful graft, not only can cell survival be impaired but the MSC's differentiation may be altered in an unknown way.

Another difficulty is establishing which patients are candidates for MSC therapy. A patient with a Thompson of 4-5 most likely would not be a candidate due to the extreme microenvironment [67]. It should be considered that 20–50% of asymptomatic patients have radiological signs of IVD degeneration raising the question of the timing of the treatment [74]. Early treatment may perhaps be the difference from symptom relief and failed therapy, regardless of cell survival and proliferation. Two clinical trials show different results on symptomatic relief in patients with IVD degeneration after undergoing stem-cell transplantation. A Thompson score of 2-3 might be the ideal candidate for MSC therapy, but this remains to be studied.

Combination therapy, providing supportive matrix and bioactive substances, may possibly be the best treatment required, optimizing cell survival, proliferation, and differentiation [55, 74]. Several growth factors described in previous studies have been implicated in IVD degeneration and therapy. MSCs secreting transforming growth factor-beta (TGF-*β*), Insulin-like growth factor-1 (IGF-1), and platelet-derived growth factor (PDGF) have been found in cocultures with NP cells and have been shown to be an effective stimulator on matrix metabolism and cell proliferation during biological repair of IVDs [13, 67]. Growth and differentiation factor-5 has been shown to increase disc height and stimulate proliferation and matrix synthesis in the NP and AF. Furthermore, Henriksson et al. found endogenous stem cell niches in the AF border to the ligament zone and the perichondrium region [21]. The utilization of growth factors may stimulate proliferation of these endogenous stem cells. It is reasonable to assume that injection of naked growth factors within the scaffold containing the MSCs at time of transplantation may increase graft survival and cell proliferation and differentiation into NP. Bringing into question what type of scaffold if any is the most adequate for transplantation. Immunogenicity, architectural and mechanical properties along with biocompatibility, biodegradability, and method of graft delivery need to be considered when choosing the scaffold [76]. Dosing studies will also need to be done in order to determine the cell density and volume that will need to be transplanted in order to obtain the desired effect while causing the least amount of side effects. Moreover, to be determined will be the need for subsequent treatments or if one time treatment will suffice.

Given that the IVD is considered immunoprivileged, the need to find an autologous cell origin might not be necessary [7, 67]. Although this should be studied further to ascertain if an immunosuppressive regimen will be needed and for how long.

One last consideration is the ideal culture conditions of the MSCs. First of all, in order to be used for clinical trials it must be done in GMP grade conditions with xeno-free reagents. Coculture of NP cells with MCS may be necessary in order to enhance the biological and metabolic viability of the cells [67]. It is important to consider that *in vitro* expansion can lead to an accumulation of genetic and epigenetic changes with an unknown effect *in vivo* once transplanted. The changes may lead to increased immunogenicity even when autologous or malignant transformation.

## 6. Conclusion

It is evident that there are many questions left unanswered. In order to move forward in finding an effective therapeutic option for IVD degeneration-associated back pain, they will need to be studied further. One of the main obstacles is creating an animal model that can adequately replicate the microenvironment seen in IVD degeneration. Once an animal model is established, more preclinical data will be able to be collected in a directed way with adequate conditions.

## Figures and Tables

**Table 1:** Review of stem cell intervertebral disc therapy reported in the literature, including the animal model, cell type, and treatment outcome.

Study year	Animal model	Cell type	Cellular scaffold	Result
Nishimura and Mochida, 1998 [41]	Rat (nucleus aspiration)	Autologous NP tissue	NA	NA
Okuma et al., 2000 [42]	Rabbit (nucleus aspiration)	NP cells	No	Delayed formation of clusters of chondrocyte-like cells, the destruction of disc architecture, and the elaboration of type-II collagen
Nomura et al., 2001 [43]	Rabbit (nucleus aspiration)	Allograft NP tissue	No	Decreased IVD
Gruber et al. 2002 [77]	Sand rat	Autologous disc chondrocytes—AF cells	No	Engrafted cells integrated into the disc and normal ECM was synthetized
Ganey et al., 2003 [45]	Canine (disc material removal)	Autologous disc chondrocytes—NP and AF cells	No	Viable proliferating chondrocytes that synthetized ECM (collagen I and II) were found and retention of disc height
Gorensek et al., 2004 [46]	Rabbit (nucleus aspiration)	Autologous cartilage chondrocytes	No	Only Hyaline-like cartilage was found
Sakai et al. 2003 [51], Sakai et al. 2005 [78]	Rabbit (nucleus aspiration)	Autologous BMSCs—genetic marking with LacZ	Atelocollagen gel	Improved annular structure and proteoglycan preservation
Crevensten et al., 2004 [47]	Rat (no injury)	BMSCs	Hyaluronan gel	Increased disc height and matrix synthesis
Sakai et al. 2005 [78]	Rabbit (nucleus aspiration)	Autologous BMSCs—genetic marking with GFP	Atelocollagen gel	Proliferation and site-dependent differentiation
Zhang et al., 2005 [55]	Rabbit (no injury)	Allogeneic BMSCs—genetic marking with LacZ	No	Increased proteoglycan and collagen type II synthesis
Leung et al. 2006 [79]	Rabbit (nucleus puncture)	Allogeneic BMSCs	NA	NA
Sobajima et al., 2008 [52]	Rabbit (no injury)	Allogeneic BMSCs—genetic marking with LacZ	No	Transplanted BMSCs migration and engraftment into the inner annulus fibrosus
Hiyama et al., 2008 [49]	Canine (nucleotomy)	Autologous BMSCs	No	Suppression of disc degeneration and preservation of immune privilege
Hoogendoorn et al. 2008 [80]	Goat (ABC chondroitinase)	None		Mild slowly progressive degeneration
Yang et al., 2009 [54]	Murine (annular puncture)	BMSCs from EGFP transgenic mice	No	Increased matrix synthesis by both autonomous differentiation and stimulatory action on endogenous cells
Henriksson et al., 2009 [48]	Porcine (nucleus aspiration)	Human BMSCs	Hydrogel	Cells survival and disc-like differentiation
Wei et al., 2009 [53]	Rat (no injury)	Human BMSCs labeled with tracker orange	No	Cells survival and chondrocytic differentiation
Acosta et al., 2011 [44]	Porcine (nucleotomy)	Juvenile Chondrocytes/Allogeneic BMSCs	Fibrin	JC survival, proliferation, and synthesis of ECM. MSCs were not observed

NP: nucleus pulposus, AF: annulus fibrosus, ECM: extracellular matrix, MSCs: mesenchymal stem cells, BMSCs: bone marrow mesenchymal stem cells, and IVD: intervertebral disc.

## References

[1] Buckwalter JA (1995). Spine update: aging and degeneration of the human intervertebral disc. *Spine*.

[2] Haefeli M, Kalberer F, Saegesser D, Nerlich AG, Boos N, Paesold G (2006). The course of macroscopic degeneration in the human lumbar intervertebral disc. *Spine*.

[3] Luoma K, Riihimäki H, Luukkonen R, Raininko R, Viikari-Juntura E, Lamminen A (2000). Low back pain in relation to lumbar disc degeneration. *Spine*.

[4] Pye SR, Reid DM, Smith R (2004). Radiographic features of lumbar disc degeneration and self-reported back pain. *Journal of Rheumatology*.

[5] Deyo RA, Mirza SK, Martin BI (2006). Back pain prevalence and visit rates: estimates from U.S. national surveys, 2002. *Spine*.

[6] Rubin DI (2007). Epidemiology and risk factors for spine pain. *Neurologic Clinics*.

[7] Hart LG, Deyo RA, Cherkin DC (1995). Physician office visits for low back pain: frequency, clinical evaluation, and treatment patterns from a U.S. National survey. *Spine*.

[8] Gou HR, Tanaka S, Halperin WE, Cameron LL (1999). Back pain prevalence in US industry and estimates of lost workdays. *American Journal of Public Health*.

[9] Labar G (1992). A battle plan for back injury prevention. *Occupational Hazards*.

[10] Maetzel A, Li L (2002). The economic burden of low back pain: a review of studies published between 1996 and 2001. *Best Practice and Research: Clinical Rheumatology*.

[11] Manchikanti L (2000). Epidemiology of low back pain. *Pain Physician*.

[12] Rizzo JA, Abbott TA, Berger ML (1998). The Labor Productivity Effects of Chronic Backache in the United States. *Medical Care*.

[13] Van Tulder MW, Koes BW, Bouter LM (1995). A cost-of-illness study of back pain in The Netherlands. *Pain*.

[14] Freburger JK, Holmes GM, Agans RP (2009). The rising prevalence of chronic low back pain. *Archives of Internal Medicine*.

[15] Boos N, Weissbach S, Rohrbach H, Weiler C, Spratt KF, Nerlich AG (2002). Classification of age-related changes in lumbar intervertebral discs: 2002 Volvo award in basic science. *Spine*.

[16] Antoniou J, Steffen T, Nelson F (1996). The human lumbar intervertebral disc: evidence for changes in the biosynthesis and denaturation of the extracellular matrix with growth, maturation, ageing, and degeneration. *Journal of Clinical Investigation*.

[17] Freemont TJ, LeMaitre C, Watkins A, Hoyland JA (2001). Degeneration of intervertebral discs: current understanding of cellular and molecular events, and implications for novel therapies. *Expert Reviews in Molecular Medicine*.

[18] Adams MA, Freeman BJC, Morrison HP, Nelson IW, Dolan P (2000). Mechanical initiation of intervertebral disc degeneration. *Spine*.

[19] Horner HA, Urban JPG (2001). 2001 Volvo award winner in basic science studies: effect of nutrient supply on the viability of cells from the nucleus pulposus of the intervertebral disc. *Spine*.

[20] Nachemson A, Lewin T, Maroudas A, Freeman MA (1970). In vitro diffusion of dye through the end-plates and the annulus fibrosus of human lumbar inter-vertebral discs. *Acta Orthopaedica Scandinavica*.

[21] Handa T, Ishihara H, Ohshima H, Osada R, Tsuji H, Obata K (1997). Effects of hydrostatic pressure on matrix synthesis and matrix metalloproteinase production in the human lumbar intervertebral disc. *Spine*.

[22] Brown MD, Tsaltas TT (1976). Studies on the permeability of the intervertebral disc during skeletal maturation. *Spine*.

[23] Bibby SRS, Urban JPG (2004). Effect of nutrient deprivation on the viability of intervertebral disc cells. *European Spine Journal*.

[24] Ishihara H, Urban JPG (1999). Effects of low oxygen concentrations and metabolic inhibitors on proteoglycan and protein synthesis rates in the intervertebral disc. *Journal of Orthopaedic Research*.

[25] Urban JPG, Holm S, Maroudas A, Nachemson A (1977). Nutrition of the intervertebral disk. An in vivo study of solute transport. *Clinical Orthopaedics and Related Research*.

[26] Le Maitre CL, Freemont AJ, Hoyland JA (2004). Localization of degradative enzymes and their inhibitors in the degenerate human intervertebral disc. *Journal of Pathology*.

[27] Roberts S, Caterson B, Menage J, Evans EH, Jaffray DC, Eisenstein SM (2000). Matrix metalloproteinases and aggrecanase: their role in disorders of the human intervertebral disc. *Spine*.

[28] Slade SC, Keating JL (2007). Unloaded movement facilitation exercise compared to no exercise or alternative therapy on outcomes for people with nonspecific chronic low back pain: a systematic review. *Journal of Manipulative and Physiological Therapeutics*.

[29] Yang X, Li X (2009). Nucleus pulposus tissue engineering: a brief review. *European Spine Journal*.

[30] Fritzell P, Hägg O, Wessberg P, Nordwall A (2001). 2001 Volvo award winner in clinical studies: lumbar fusion versus nonsurgical treatment for chronic low back pain. A multicenter randomized controlled trial from the Swedish Lumbar Spine Study Group. *Spine*.

[31] Gillet P (2003). The fate of the adjacent motion segments after lumbar fusion. *Journal of Spinal Disorders and Techniques*.

[32] Hambly MF, Wiltse LL, Raghavan N, Schneiderman G, Koenig C (1998). The transition zone above a lumbosacral fusion. *Spine*.

[33] Schlegel JD, Smith JA, Schleusener RL (1996). Lumbar motion segment pathology adjacent to thoracolumbar, lumbar, and lumbosacral fusions. *Spine*.

[34] Acosta FL, Lotz J, Ames CP (2005). The potential role of mesenchymal stem cell therapy for intervertebral disc degeneration: a critical overview. *Neurosurgical Focus*.

[35] Masuda K, Oegema TR, An HS (2004). Growth factors and treatment of intervertebral disc degeneration. *Spine*.

[36] An HS, Takegami K, Kamada H (2005). Intradiscal administration of osteogenic protein-1 increases intervertebral disc height and proteoglycan content in the nucleus pulposus in normal adolescent rabbits. *Spine*.

[37] Li X, Leo BM, Beck G, Balian G, Anderson DG (2004). Collagen and proteoglycan abnormalities in the GDF-5-deficient mice and molecular changes when treating disk cells with recombinant growth factor. *Spine*.

[38] Thompson JP, Oegema TR, Bradford DS (1991). Stimulation of mature canine intervertebral disc by growth factors. *Spine*.

[39] Walsh AJL, Bradford DS, Lotz JC (2004). In Vivo growth factor treatment of degenerated intervertebral discs. *Spine*.

[40] Yoon ST, Kim KS, Li J (2003). The effect of bone morphogenetic protein-2 on rat intervertebral disc cells in vitro. *Spine*.

[41] Nishimura K, Mochida J (1998). Percutaneous reinsertion of the nucleus pulposus: an experimental study. *Spine*.

[42] Okuma M, Mochida J, Nishimura K, Sakabe K, Seiki K (2000). Reinsertion of stimulated nucleus pulposus cells retards intervertebral disc degeneration: an in vitro and in vivo experimental study. *Journal of Orthopaedic Research*.

[43] Nomura T, Mochida J, Okuma M, Nishimura K, Sakabe K (2001). Nucleus pulposus allograft retards intervertebral disc degeneration. *Clinical Orthopaedics and Related Research*.

[44] Acosta FL, Metz L, Adkisson HD (2011). Porcine intervertebral disc repair using allogenic juvenile articular chondrocytes or mesenchymal stem cells. *Tissue Engineering*.

[45] Ganey T, Libera J, Moos V (2003). Disc chondrocyte transplantation in a canine model: a treatment for
degenerated or damaged intervertebral disc. *Spine*.

[46] Gorensek M, Joksimovic C, Kregar-Velikonja N (2004). Nucleus pulposus repair with cultured autologous elastic cartilage derived chondrocytes. *Cellular and Molecular Biology Letters*.

[47] Crevensten G, Walsh AJL, Ananthakrishnan D (2004). Intervertebral disc cell therapy for regeneration: mesenchymal stem cell implantation in rat intervertebral discs. *Annals of Biomedical Engineering*.

[48] Henriksson HB, Svanvik T, Jonsson M (2009). Transplantation of human mesenchymal stems cells into intervertebral discs in a xenogeneic porcine model. *Spine*.

[49] Hiyama A, Mochida J, Iwashina T (2008). Transplantation of mesenchymal stem cells in a canine disc degeneration model. *Journal of Orthopaedic Research*.

[50] Ho G, Leung VYL, Cheung KMC, Chan D (2008). Effect of severity of intervertebral disc injury on mesenchymal stem cell-based regeneration. *Connective Tissue Research*.

[51] Sakai D, Mochida J, Yamamoto Y (2003). Transplantation of mesenchymal stem cells embedded in Atelocollagen gel to the intervertebral disc: a potential therapeutic model for disc degeneration. *Biomaterials*.

[52] Sobajima S, Vadala G, Shimer A, Kim JS, Gilbertson LG, Kang JD (2008). Feasibility of a stem cell therapy for intervertebral disc degeneration. *Spine Journal*.

[53] Wei A, Tao H, Chung SA, Brisby H, Ma DD, Diwan AD (2009). The fate of transplanted xenogeneic bone marrow-derived stem cells in rat intervertebral discs. *Journal of Orthopaedic Research*.

[54] Yang F, Leung VYL, Luk KDK, Chan D, Cheung KMC (2009). Mesenchymal stem cells arrest intervertebral disc degeneration through chondrocytic differentiation and stimulation of endogenous cells. *Molecular Therapy*.

[55] Zhang YG, Guo X, Xu P, Kang LL, Li J (2005). Bone mesenchymal stem cells transplanted into rabbit intervertebral discs can increase proteoglycans. *Clinical Orthopaedics and Related Research*.

[56] Pittenger MF, Mackay AM, Beck SC (1999). Multilineage potential of adult human mesenchymal stem cells. *Science*.

[57] Jiang Y, Jahagirdar BN, Reinhardt RL (2002). Pluripotency of mesenchymal stem cells derived from adult marrow. *Nature*.

[58] Minguell JJ, Erices A, Conget P (2001). Mesenchymal stem cells. *Experimental Biology and Medicine*.

[59] Frankel MS (2000). In search of stem cell policy. *Science*.

[60] McLaren A (2001). Ethical and social considerations of stem cell research. *Nature*.

[61] Pittenger MF, Martin BJ (2004). Mesenchymal stem cells and their potential as cardiac therapeutics. *Circulation Research*.

[62] Shake JG, Gruber PJ, Baumgartner WA (2002). Mesenchymal stem cell implantation in a swine myocardial infarct model: engraftment and functional effects. *Annals of Thoracic Surgery*.

[63] Bang OY, Lee JS, Lee PH, Lee G (2005). Autologous mesenchymal stem cell transplantation in stroke patients. *Annals of Neurology*.

[64] Kuo TK, Hung SP, Chuang CH (2008). Stem cell therapy for liver disease: parameters governing the success
of using bone marrow mesenchymal stem cells. *Gastroenterology*.

[65] De Bari C, Dell’Accio F, Luyten FP (2001). Human periosteum-derived cells maintain phenotypic stability and chondrogenic potential throughout expansion regardless of donor age. *Arthritis and Rheumatism*.

[66] De Bari C, Dell’Accio F, Tylzanowski P, Luyten FP (2001). Multipotent mesenchymal stem cells from adult human synovial membrane. *Arthritis and Rheumatism*.

[67] Zuk PA, Zhu M, Ashjian P (2002). Human adipose tissue is a source of multipotent stem cells. *Molecular Biology of the Cell*.

[68] Sakaguchi Y, Sekiya I, Yagishita K, Muneta T (2005). Comparison of human stem cells derived from various mesenchymal tissues: superiority of synovium as a cell source. *Arthritis and Rheumatism*.

[69] Yoshimura H, Muneta T, Nimura A, Yokoyama A, Koga H, Sekiya I (2007). Comparison of rat mesenchymal stem cells derived from bone marrow, synovium, periosteum, adipose tissue, and muscle. *Cell and Tissue Research*.

[70] Takada T, Nishida K, Doita M, Kurosaka M (2002). Fas ligand exists on intervertebral disc cells: a potential molecular mechanism for immune privilege of the disc. *Spine*.

[71] Bartholomew A, Sturgeon C, Siatskas M (2002). Mesenchymal stem cells suppress lymphocyte proliferation in vitro and prolong skin graft survival in vivo. *Experimental Hematology*.

[72] Masuda K, Aota Y, Muehleman C (2005). A novel rabbit model of mild, reproducible disc degeneration by an anulus needle puncture: correlation between the degree of disc injury and radiological and histological appearances of disc degeneration. *Spine*.

[73] Benneker LM, Heini PF, Alini M, Anderson SE, Ito K (2005). 2004 Young investigator award winner: vertebral endplate marrow contact channel occlusions and intervertebral disc degeneration. *Spine*.

[74] Brisby H, Tao H, Ma DDF, Diwan AD (2004). Cell therapy for disc degeneration - Potentials and pitfalls. *Orthopedic Clinics of North America*.

[75] Wakitani S, Imoto K, Yamamoto T, Saito M, Murata N, Yoneda M (2002). Human autologous culture expanded bone marrow-mesenchymal cell transplantation for repair of cartilage defects in osteoarthritic knees. *Osteoarthritis and Cartilage*.

[76] Zhang Y, An HS, Tannoury C, Thonar EJMA, Freedman MK, Anderson DG (2008). Biological treatment for degenerative disc disease: implications for the field of physical medicine and rehabilitation. *American Journal of Physical Medicine and Rehabilitation*.

[77] Gruber HE, Johnson TL, Leslie K (2002). Autologous intervertebral disc cell implantation: A model using Psammomys obesus, the sand rat. *Spine*.

[78] Sakai D, Mochida J, Iwashina T (2005). Differentiation of mesenchymal stem cells transplanted to a rabbit degenerative disc model: Potential and limitations for stem cell therapy in disc regeneration. *Spine*.

[79] Leung VYL, Chan D, Cheung KMC (2006). Regeneration of intervertebral disc by mesenchymal stem cells: Potentials, limitations, and future direction. *European Spine Journal*.

[80] Hoogendoorn RJW, Helder MN, Kroeze RJ, Bank RA, Smit TH, Wuisman PIJ (2008). Reproducible long-term disc degeneration in a large animal model. *Spine*.

